# A novel 33‐Gene targeted resequencing panel provides accurate, clinical‐grade diagnosis and improves patient management for rare inherited anaemias

**DOI:** 10.1111/bjh.14221

**Published:** 2016-07-19

**Authors:** Noémi B. A. Roy, Edward A. Wilson, Shirley Henderson, Katherine Wray, Christian Babbs, Steven Okoli, Wale Atoyebi, Avery Mixon, Mary R. Cahill, Peter Carey, Jonathan Cullis, Julie Curtin, Helene Dreau, David J. P. Ferguson, Brenda Gibson, Georgina Hall, Joanne Mason, Mary Morgan, Melanie Proven, Amrana Qureshi, Joaquin Sanchez Garcia, Nongnuch Sirachainan, Juliana Teo, Ulf Tedgård, Doug Higgs, David Roberts, Irene Roberts, Anna Schuh

**Affiliations:** ^1^ BRC Blood Theme and BRC/NHS Translational Molecular Diagnostics CentreJohn Radcliffe HospitalOxfordUK; ^2^ Molecular Haematology UnitWeatherall Institute of Molecular MedicineJohn Radcliffe HospitalOxfordUK; ^3^ Department of HaematologyOxford University Hospitals NHS Foundation TrustChurchill HospitalOxfordUK; ^4^ Division of Pediatric Hematology/OncologyChildren's Hospital at ErlangerChattanoogaTNUSA; ^5^ Department of HaematologyCork University HospitalCorkIreland; ^6^ Department of HaematologyThe Royal Victoria InfirmaryNewcastle‐upon‐TyneUK; ^7^ Department of HaematologySalisbury NHS Foundation TrustSalisburyUK; ^8^ Department of HaematologySydney Children's Hospitals NetworkWestmeadAustralia; ^9^ Nuffield Department of Clinical Laboratory SciencesJohn Radcliffe HospitalUniversity of OxfordOxfordUK; ^10^ Department of Paediatric Haematology/OncologyRoyal Hospital for ChildrenGlasgowUK; ^11^ Paediatric Haematology/Oncology UnitOxford University Hospitals NHS Foundation TrustJohn Radcliffe HospitalOxfordUK; ^12^ Department of Paediatric Haematology‐OncologyUniversity Hospital SouthamptonSouthamptonUK; ^13^ Laboratorio Diagnóstico UGC de Hematología Hospital Universitario Reina SofíaCórdobaSpain; ^14^ Division of Haemato‐OncologyDepartment of PaediatricsFaculty of MedicineRamathibodi HospitalMahidol UniversityBangkokThailand; ^15^ Department of PaediatricsSkåne University HospitalLundSweden; ^16^ NHS Blood and TransplantNHSBT – John Radcliffe Hospital, Level 2OxfordUK

**Keywords:** inherited anaemia, congenital dyserythropoietic anaemia, molecular genetics, pyruvate kinase deficiency, next‐generation sequencing

## Abstract

Accurate diagnosis of rare inherited anaemias is challenging, requiring a series of complex and expensive laboratory tests. Targeted next‐generation‐sequencing (NGS) has been used to investigate these disorders, but the selection of genes on individual panels has been narrow and the validation strategies used have fallen short of the standards required for clinical use. Clinical‐grade validation of negative results requires the test to distinguish between lack of adequate sequencing reads at the locations of known mutations and a real absence of mutations. To achieve a clinically‐reliable diagnostic test and minimize false‐negative results we developed an open‐source tool (CoverMi) to accurately determine base‐coverage and the ‘discoverability’ of known mutations for every sample. We validated our 33‐gene panel using Sanger sequencing and microarray. Our panel demonstrated 100% specificity and 99·7% sensitivity. We then analysed 57 clinical samples: molecular diagnoses were made in 22/57 (38·6%), corresponding to 32 mutations of which 16 were new. In all cases, accurate molecular diagnosis had a positive impact on clinical management. Using a validated NGS‐based platform for routine molecular diagnosis of previously undiagnosed congenital anaemias is feasible in a clinical diagnostic setting, improves precise diagnosis and enhances management and counselling of the patient and their family.

Diagnosis of the cause of anaemia is usually straightforward using simple laboratory tests (e.g. assessment of iron status, haemoglobin high performance liquid chromatography). In contrast, for patients with uncommon or rare congenital anaemias, many of which are severe, reaching a precise diagnosis is often extremely difficult and may take several years (Magor *et al*, 2005; Paessler & Hartung, 2015; Van Zwieten *et al*, 2015). With the exception of red cell membrane disorders, red cell morphology is often non‐specific (Koralkova *et al*, 2014) and may require exceptional expertise; many investigations are expensive and restricted to specialist laboratories (Grace *et al*, 2015). Thus, it is unsurprising that many patients with rare congenital anaemias have no precise diagnosis, which prevents effective patient management and genetic counselling.

Eventually, whole‐genome sequencing (WGS) may become a realistic diagnostic tool to be used early in the investigation of inherited conditions and has recently shown promising results in a study of critically ill neonates with suspected genetic diagnoses 57% (Petrikin *et al*, 2015). However, despite increasing accessibility and cost effectiveness of sequencing technology and analysis (Meienberg *et al*, 2016), interpretation of the data remains complex and the overall utility of WGS in a clinical context is still being investigated. Meanwhile, targeted next‐generation‐sequencing (NGS) is a popular approach for providing rapid and accurate mutation analysis as the use of a limited panel of genes facilitates the interpretation and reduces incidental findings (Sun *et al*, 2015). While targeted NGS has been reported for the investigation of various anaemias and/or pancytopenia (Gerrard *et al*, 2013; Collopy *et al*, 2014; De Rocco *et al*, 2014; Christensen *et al*, 2015; Ghemlas *et al*, 2015; Zhang *et al*, 2015), these studies have reported only research‐grade validation strategies, usually by ensuring that a small number of variants or single nucleotide polymorphisms (SNPs; ranging from 2 to 53 variants) identified by Sanger sequencing could also be detected by NGS. While these panels are valuable in the research context for the positive identification of novel mutations in known genes, they cannot be reliably used in a clinical context because ‘negative’ results do not distinguish between the absence of mutations on the one hand and lack of adequate sequencing reads at the locations of known mutations on the other. This is now essential for medical laboratory accreditation in some countries, including the UK.

Here, we report the development and application of a novel clinical‐grade open‐source tool for evaluation of ‘per‐base coverage’ in a 33 gene‐targeted NGS panel (Oxford Red Cell Panel, ORCP) used to provide a clinical diagnosis to patients with unexplained anaemia. Specifically, we aimed to (i) demonstrate the feasibility of achieving clinical‐grade validation of a targeted re‐sequencing panel for congenital anaemias; (ii) develop a clinical‐grade open‐source tool for evaluation of read coverage; (iii) assess the diagnostic yield of this strategy for congenital anaemias and (iv) assess the clinical impact of results generated using this approach. To select the genes for inclusion in the ORCP we chose to take a relatively unbiased approach by including causative genes for conditions where anaemia may be part of a wider bone marrow failure syndrome as well as those for specific red cell disorders, such as Diamond–Blackfan Anaemia (DBA) and Congenital Dyserythropoietic Anaemia (CDA).

## Materials and methods

The workflow from sample preparation to clinical interpretation is shown in Fig S1. In most cases, targeted NGS was only performed after standard investigations, such as erythrocyte adenine deaminase (eADA) levels and chromosomal breakage studies, had been performed. All additional tests performed are included in Table SI. Patients with microcytic hypochromic indices were tested for haemoglobinopathies by direct sequencing of the *HBA1/2* and *HBB* genes and multiplex gap‐polymerase chain reaction (PCR) to exclude the common 3·7 and 4·2 kb single *HBA1/2* gene deletions. Multiplex ligation‐dependent probe amplification (MLPA) was undertaken where appropriate to investigate the presence of other deletions or duplications of *HBA1/2* or *HBB*.

The study was approved by the Wales Research Ethics Committee (REC5) (13/WA/0371) with written consent from patients and/or parents.

### Design of the ORCP (Oxford Red Cell Panel)

Genes were selected from the literature and the Human Gene Mutation Database (HGMD^®^; http://www.biobase-international.com/product/hgmd) as causative for the anaemias under study (Table 1, Table SII). illumina designstudio v1.0 was used to design the TruSeq Custom Amplicon panel (TSCA) (both Illumina, San Diego, USA) to 249 specified regions using 425 bp amplicons with UCSC hg19 (https://genome.ucsc.edu/cgi-bin/hgTracks?db=hg19&lastVirtModeType=default&lastVirtModeExtraState=&virtModeType=default&virtMode=0&nonVirtPosition=&position=chr21%3A33031597-33041570&hgsid=498013795_KW6GKVQmp3e84XkCP3AAdx93cKft) as the reference genome. A total of 498 amplicons were designed to capture exons and intron/exon boundaries. Promoter regions were included in the design.

**Table 1:** Genes included on the Oxford red cell panel

Condition	Genes
Congenital Dyserythropoietic Anaemia (CDA)	*C15ORF41, CDAN1, SEC23B, KIF23, KLF1*
Diamond–Blackfan Anaemia (DBA)	*RPL11, RPL26, RPL35A, RPL5, RPS10, RPS17, RPS19, RPS24, RPS7, RPL19, RPL27, RPL9, RPS26, RPS29, RPS27, GATA1*
Schwachman‐Diamond Syndrome (SDS)	*SBDS*
Dyskeratosis Congenita (DKC)	*DKC1, NHP2, NOP10, TERT, TERC, TINF2*
Sideroblastic Anaemia	*ALAS2, SLC25A38*
Red cell enzyme deficiencies	*G6PD, PKLR, NT5C3A*

### Library preparation and sequencing

Library preparation was performed with illumina's tsca v1.5 kit (FC‐130‐1001) using 250 ng genomic DNA, following manufacturer's instructions. Samples were pooled (average 26 samples) and loaded at 20 pM on MiSeq using a v3 600‐cycle reagent kit sequencing 2x301 paired‐end reads (Illumina).

### Sanger sequencing

Sequencing was performed on an ABI3730 DNA Analyser (Applied Biosystems, Foster City, CA, USA) using 200–500 ng DNA template, BigDye (Applied Biosystems) reaction mix, and 3·2pmol sequencing primer. Chromatograms were visualized with sequencher v4.8. (Gene Codes Corp, Ann Arbor MI, USA).

### MPLA and SNP array

Given that ~20% of cases of DBA (Farrar *et al*, 2011) are caused by ribosomal protein gene deletions, a commercial MLPA kit was used to detect deletions in the following genes: *RPL11*,* RPL35A*,* RPS17*,* RPS19*,* RPS26*, and *RPL5* (MRC Holland, Amsterdam, the Netherlands). Infinium Human OmniExpress Exome v1.2 beadchips (Illumina) were run using 200 ng DNA following the manufacturer's protocol for Infinuim HD assay super manual workflow and immediately scanned using Illumina iScan. Data were analysed using illumina genomestudio (v2011.1).

### Bioinformatics and mutation calling

Sequencing reads were aligned and variants called against reference genome (hg19) using the miseq reporter software (v2.2.29·0, Illumina) and the TSCA panel manifest. Variants were annotated and filtered using variantstudio (v2.2, Illumina). Variant filters were established using a sample set with known mutations and filter settings verified using blinded samples: quality score >100; passing filter; total read frequency of >30, mutant allele frequency of ≥20%; variant present in <5% of the population; of consequence– missense, frameshift, stop gained, stop lost, initiator codon, in‐frame insertion, in‐frame deletion, splice. Filtered variants were visualized in genomic context using IGV (Interactive Genomics Viewer: http://www.broadinstitute.org/igv/) to exclude sequencing artifacts.

### CoverMi

To report coverage/base and discoverability of all known variants we developed an open‐source tool, CoverMi, written in Python (v2.7.6) and R (v3.1.2) and run on Windows, Linux and OSX. It utilizes Bedtools (v2.221, http://bedtools.readthedocs.org) to extract raw coverage information from the alignment.bam file and maps this to the gene, exon and variant locations on the UCSC hg19 reference genome. CoverMi has a simple graphical user interface designed for use by laboratory scientists and clinicians without extensive bioinformatics knowledge. CoverMi can be used with any sequencing data generated by Illumina or ThermoFisher platforms; it is licensed under an MIT open‐source license and is freely available for download at https://github.com/eawilson/covermi.

The American College of Medical Genetics (ACMG) guidelines were used for reporting of genetic variants (Rehm *et al*, 2013): *Tier 1*‐ variants reported in the literature, catalogued in HGMD^®^ and validated as pathogenic. *Tier 2*‐ variants *expected to cause* the disorder due to a predicted shift in mRNA reading frame, mutation of the initiation or stop codon, introduction of a novel stop codon or alteration of the highly‐conserved GT/AG nucleotides at a splice junction, but not previously described or described only in a related phenotype; and *Tier 3*‐ variants that *may or may not be causative* of the disorder. These include pathogenic mutations in genes not normally thought to cause this particular phenotype or those predicted to produce a cryptic splice site, to affect transcription, or non‐conservative missense mutations with a high deleterious predictive score (e.g. by algorithms such as SIFT or Polyphen2). Where it was difficult to determine pathogenicity, parental or other familial DNA was sought so that the variants could be traced to determine whether they segregate with the disease. Where two known pathogenic variants were co‐inherited, each being causative for a different autosomal recessively inherited anaemia, we did not conclude that they are pathogenic when inherited together without carrying out further functional analysis and they remain ‘unsolved’. Our laboratory follows the latest ACGM guidelines (Richards *et al*, 2015) and undertakes regular reviews to see whether any previously identified variants of uncertain significance (VUSs) have now been reported as either pathogenic or non‐pathogenic. The ORCP has undergone certification according to International Organization for Standardization (ISO) standard 015189 of the United Kingdom Accreditation Service (UKAS), the medical laboratory accreditation authority in the UK and can accept diagnostic referrals from within as well as outside the UK.

## Results

### Validation of ORCP

Validation of ORCP followed the 2013 ACMG guidelines (Rehm *et al*, 2013). *Sensitivity and specificity*: 44 unique variants identified by ORCP were confirmed by Sanger sequencing (one sequence variant previously identified by Sanger sequencing was not detected by ORCP using set filters). Using the array comparative genomic hybridization microarray platform, comparison of the 283/273,000 features overlapping with ORCP amplicons (16 variants; 267 reference calls) showed 100% concordance, indicating 100% specificity and 99·7% sensitivity (95% confidence interval 97·9–99·9%). Of the 1207 variants in the 33‐gene panel described in HGMD^®^, 87% are missense changes, 4% simple insertions, 8% deletions and 1% complex insertions/deletions (indels). Of the 44 variants tested, 11% were insertions and 8% deletions, satisfying ACMG guidelines that positive controls include variants representative of naturally‐occurring disease‐causing traits. *Robustness and assay precision*: To test within‐run and between‐run variability, we took a much more stringent approach than used in research‐grade targeted NGS panels (Gerrard *et al*, 2013; Collopy *et al*, 2014; De Rocco *et al*, 2014; Christensen *et al*, 2015; Ghemlas *et al*, 2015; Zhang *et al*, 2015) by assaying 13 samples in duplicate and 4 in triplicate (66 variants in total, 100% of which were found on repeat sequencing).

### An open‐source tool (CoverMi) reports coverage‐per‐base and ‘discoverability’ of known variants

**Figure 1:**
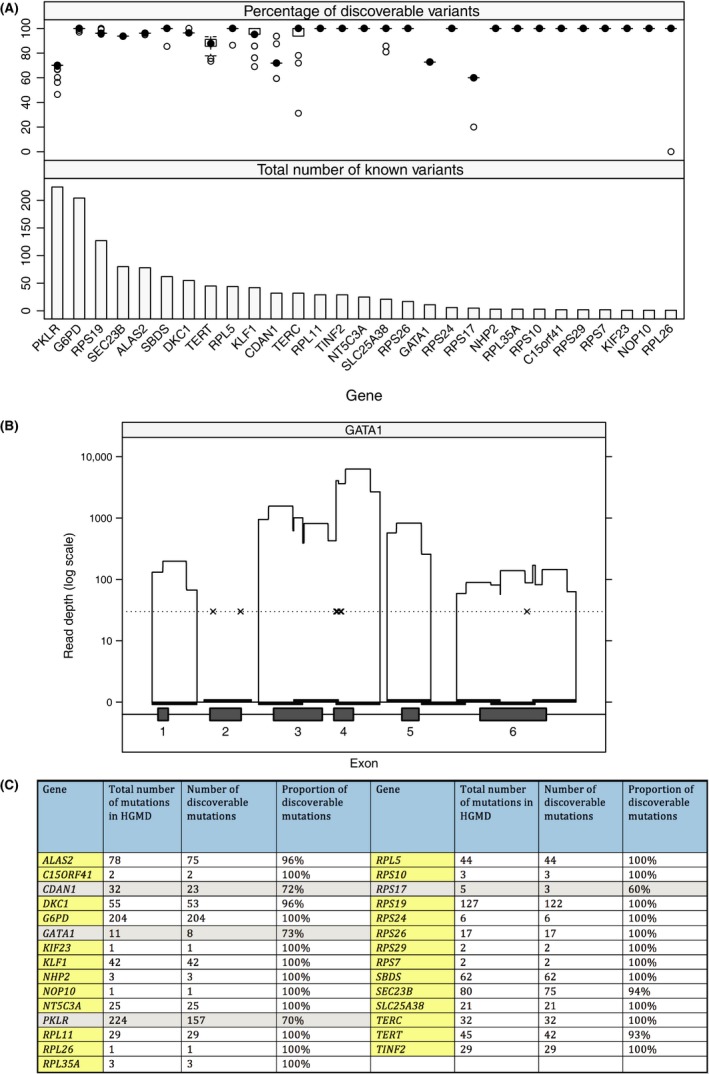
Discoverability of individual mutations across different genes on the Oxford Red Cell Panel using CoverMi software (A) Top panel: Box and whisker plot showing the percentage of known variants that are discoverable (coverage depth >30) for 72 samples over 5 separate MiSeq runs. Bottom panel shows the total number of variants for each corresponding gene as reported in the Human Gene Mutation Database (HGMD^®^). Genes not depicted on this figure do not have any variants reported in HGMD^®^. (B) Graphical representation of coverage over the *GATA1* gene for a single sample. The six exons that comprise the *GATA1* gene are displayed along the bottom of the graph and the overlapping black bars represent the ten amplicons that cover this gene. The minimum read depth for reliable detection is represented by the dotted line, along which crosses mark the locations of the known variants in each exon. It can be seen that there is adequate coverage over eight of the ten amplicons, however the second and seventh amplicons have failed. The seventh amplicons covers an intergenic region with no known variants, but the second amplicon covers exon 2, which contains two known variants. Loss of coverage over this region will increase the false negative rate of the assay in this particular sample. (C) Individual mutation coverage summary per gene per sample. For each sample on each run, CoverMi determines the percentage of known mutations in each of the genes tested that would be discoverable in that sample, by calculating the number of reads at individual nucleotide positions corresponding to previously described mutations. Where this ratio is <90%, the gene is highlighted in grey to indicate that overall coverage falls below accepted standards.

Available commercial analysis software may incorrectly label variants as ‘discoverable’ as they report mean coverage per whole amplicon, potentially masking areas of low coverage flanking central overlapping regions between forward and reverse reads (Fig S2). We therefore developed CoverMi to accurately calculate the sensitivity of ORCP for each sample by measuring precise read depth at each variant location. The performance of ORCP for 1207 known variants in 33 genes was tested on 72 samples over 5 MiSeq runs (minimum coverage depth 30x). ~90% of variants were detectable for each sample (range 84–92%); most genes had consistent coverage (e.g. *SEC23B*,* RPL11*) but a small number (e.g. *PKLR*,* TERC*) showed variability between samples (Fig 1A). The importance of coverage assessment is illustrated in Fig 1B, which shows good coverage of exons 4 and 6 which contain known variants, but failure of the exon 2 amplicon means the presence or absence of known mutations in this exon cannot be determined for this sample. Reports of variant coverage are generated for each sample per run (Fig 1C) and used to guide decisions to repeat NGS or use alternative strategies, such as Sanger sequencing, e.g. where key variants that account for a high proportion of cases are not discoverable.

### Diagnostic yield and clinical impact of ORCP for congenital anaemia

**Figure 2:**
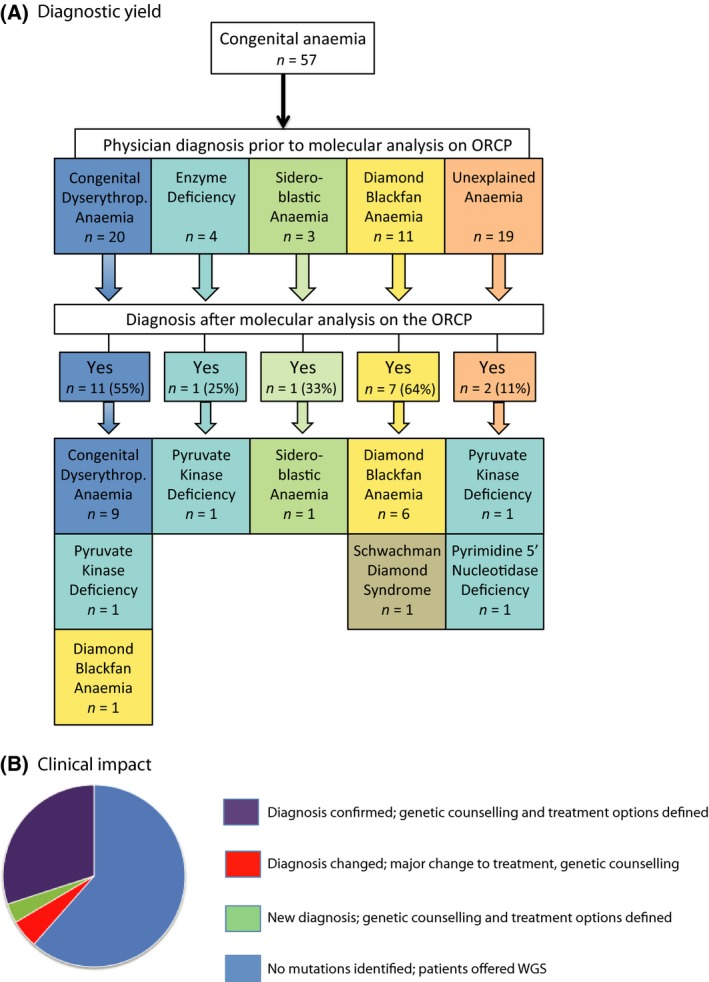
Diagnostic yield and clinical impact of targeted NGS for congenital anaemia. (A) Breakdown of the cases by working diagnosis, showing the proportion of cases where the diagnosis was confirmed or established by targeted next generation sequencing (NGS) using the Oxford Red Cell Panel (ORCP). (B) Clinical impact of targeted NGS for diagnosis of congenital anaemia. CDA, Congenital Dyserythropoietic Anaemia; DBA, Diamond–Blackfan Anaemia; SDS, Schwachman‐Diamond Syndrome; WGS, whole genome sequencing.

Following our extensive clinical‐grade validation and the development of our CoverMi tool, 57 clinical samples were analysed using ORCP (Fig 2A, Table 2, Tables SI and SIII). The ‘working diagnosis’ submitted by treating physicians was used but further details requested where molecular results were inconsistent with this. In 22/57 cases a molecular diagnosis was made using ORCP giving a diagnostic yield of 38·6%. Of these 22 cases, 17 confirmed the clinical diagnosis, two were new diagnoses in ‘unexplained anaemia’ patients and three caused a change of diagnosis, substantially altering treatment for these five patients (Fig 2B). For the remaining 35 cases, no molecular diagnosis was made, including two patients harbouring *TERT* mutations of uncertain significance and one patient with two *SEC23B* mutations of uncertain significance (Table SIV). Further investigations of these cases are continuing, including offering WGS to the families.

**Table 2:** Clinical and haematological features of patients with congenital anaemia where the ORCP resulted in a molecular diagnosis

Patient ID	Working diagnosis	Genes affected	Amino acid change (or splice site)	Reference	Tier	Effect on diagnosis	Comments	Effect on management
Unexplained anaemia								
P9	Unexplained anaemia	*PKLR*	p.Arg510Gln p.Glu241Ter	Baronciani and Beutler (1993) Percy *et al* (2007)	1 1	New diagnosis of PK deficiency	Low PK levels confirmed. Parents shown to each carry variant allele and have borderline low PK levels.	Splenectomy → transfusion independence
P14	Unexplained anaemia	*NT5C3A*	p.Gln216Ter c.456‐1G>A	Marinaki *et al* (2001)	1	New diagnosis of Pyrimidine‐5’‐nucleotidase deficiency	Confirmed by low purine pyrimidine levels	Carrier testing Monitoring Prognostic information
Diamond–Blackfan Anaemia								
P20	Diamond–Blackfan anaemia	*RPL5*	p.Ile64LeufsTer6		2	Confirmed diagnosis of DBA	Confirmed mutation is *de novo*	Carrier testing Monitoring Prognostic information
P21	Diamond–Blackfan anaemia	*RPS19*	p.Gly120AlafsTer4		2	Confirmed diagnosis of DBA	Confirmed mutation is *de novo*	Carrier testing Monitoring Prognostic information
P22	Diamond–Blackfan anaemia	*RPL5*	p.Glu129Ter		2	Confirmed diagnosis of DBA	Confirmed mutation is *de novo*	Carrier testing Monitoring Prognostic information
P23	Diamond–Blackfan anaemia	*SBDS*	c.258 + 2T> c.258 + 2T>	Boocock *et al* (2003) Boocock *et al* (2003)	1 1	Changed diagnosis from DBA to SDS	Low faecal elastase on rechecking. Neutropenia developed	Carrier testing Monitoring Prognostic information Creon
P25	Diamond–Blackfan anaemia	*RPS26*	c.181 + 3delA		3	Confirmed diagnosis of DBA	Parental samples not available	Carrier testing Monitoring Prognostic information
P26	Diamond–Blackfan anaemia	*RPL11*	p.Tyr55Ter		2	Confirmed diagnosis of DBA	Parental samples not available	Carrier testing Monitoring Prognostic information
P30	Diamond–Blackfan anaemia	*RPL5*	p.Asp59TyrfsTer53		2	Confirmed diagnosis of DBA	Parental samples not available	Carrier testing Monitoring Prognostic information
P20	Diamond–Blackfan anaemia	*RPL5*	p.Ile64LeufsTer6		2	Confirmed diagnosis of DBA	Confirmed mutation is *de novo*	Carrier testing Monitoring Prognostic information
P21	Diamond–Blackfan anaemia	*RPS19*	p.Gly120AlafsTer4		2	Confirmed diagnosis of DBA	Confirmed mutation is *de novo*	Carrier testing Monitoring Prognostic information
Sideroblastic Anaemia								
P33	Sideroblastic anaemia	*SLC25A38*	p.Ile161TyrfsTer12 p.Ile161TyrfsTer12		2 2	Confirmed diagnosis of sideroblastic anaemia	BM consistent, sibling similarly affected and same mutation	Carrier testing Monitoring Prognostic information
Enzyme deficiency								
P37	Enzyme deficiency	*PKLR*	p.Arg510Gln p.Ile342Phe	Baronciani and Beutler (1993) Baronciani *et al* (1998)	1 1	Confirmed diagnosis of enzyme deficiency	Confirmed low PK levels.	Carrier testing Monitoring Prognostic information
Congenital Dyserythropoietic Anaemia								
P38	Unexplained anaemia	*KLF1*	p.Gly335Arg p.Thr334Arg	Viprakasit *et al* (2014) Gallienne *et al* (2012)	1 1	New diagnosis of CDA‐IV	BM shows dyserythropoiesis. Patient has high HbF (14·2%), and each parent shown to carry one allele	Genetic counselling Carrier testing Prognostic information
P39	Congenital Dyserythropoietic Anaemia	*CDAN1*	p.Pro1130Leu p.Pro1130Leu	Dgany *et al* (2002) Dgany *et al* (2002)	1 1	Confirmed diagnosis of CDA‐I	EM morphology typical for CDA‐I	Carrier testing Prognostic information
P40	Congenital Dyserythropoietic Anaemia	*CDAN1*	p.Arg1042Trp p.Arg1042Trp	Dgany *et al* (2002) Dgany *et al* (2002)	1 1	Confirmed diagnosis of CDA‐I	EM morphology typical for CDA‐I	Carrier testing Prognostic information
P44	Congenital Dyserythropoietic Anaemia	*RPS19*	p.Trp52Ter	Willig *et al* (1999)	1	Changed diagnosis from CDA‐I to DBA	EM morphology reported as “typical for CDA‐I”	Carrier testing Prognostic information
P49	Congenital Dyserythropoietic Anaemia	*CDAN1*	p.Ala944Ser p.Pro672Leu p.Ala412Pro	Dgany *et al* (2002)	3 1 3	Confirmed diagnosis of CDA‐I	EM morphology typical for CDA‐I	Carrier testing Prognostic information
P50	Congenital Dyserythropoietic Anaemia	*CDAN1*	p.Val993GlyfsTer13 p.Pro672Leu	Dgany *et al* (2002)	2 1	Confirmed diagnosis of CDA‐I	EM morphology typical for CDA‐I	Carrier testing Prognostic information
P51	Congenital Dyserythropoietic Anaemia	*CDAN1*	p.Pro51Leu p.Pro51Leu		3 3	Confirmed diagnosis of CDA‐I	EM morphology typical for CDA‐I	Carrier testing Prognostic information
P53	Congenital Dyserythropoietic Anaemia	*CDAN1*	p.Leu709del p.Leu709del		3 3	Confirmed diagnosis of CDA‐I	EM morphology typical for CDA‐I	Carrier testing Prognostic information
P54	Congenital Dyserythropoietic Anaemia	*PKLR*	p.Asn393Ser p.Ala526Glufs*3		3 2	Changed diagnosis from CDA‐I to PK deficiency	EM morphology reported as “typical for CDA‐I” Confirmed low PK levels.	Carrier testing Prognostic information Splenectomy → transfusion independence
P55	Congenital Dyserythropoietic Anaemia	*CDAN1*	p.Phe52Leu p.Glu1009LeufsTer24	Tamary *et al* (2005)	1 2	Confirmed diagnosis of CDA‐I	EM morphology typical for CDA‐I	Carrier testing Prognostic information
P56	Congenital Dyserythropoietic Anaemia	*CDAN1*	p.Pro672Leu p.Phe52Leu	Dgany *et al* (2002) Tamary *et al* (2005)	1 1	Confirmed diagnosis of CDA‐I	EM morphology typical for CDA‐I	Carrier testing Prognostic information

PK, pyruvate kinase; BM, bone marrow; DBA, Diamond–Blackfan anaemia; SDS, Schwachman Diamond Syndrome; EM, electron microscopy.

Clinical and haematological features of the 19 patients in whom a molecular diagnosis was achieved using the Oxford Red Cell Panel (ORCP).

#### Diamond–Blackfan anaemia

Of 11 cases diagnosed by referring physicians as DBA, six harboured heterozygous mutations in ribosomal protein (RP) genes predicted to be pathogenic matching their bone marrow findings. In 3/6 cases, the absence of mutations in parental samples indicated the mutations were *de novo*. An additional patient, a 16‐month‐old boy with a history of low birthweight and failure to thrive, initially presented with anaemia and a bone marrow aspirate showed markedly reduced erythropoiesis together with normal myelopoiesis. At this point his neutrophil count was normal leading to a diagnosis of DBA. Following molecular analysis using the ORCP he was found to have a homozygous known pathogenic mutation in the *SBDS* gene (c.258 + 2T) and mild neutropenia developed at around that time. Sanger sequencing confirmed both parents were carriers and a revised diagnosis of Shwachman–Diamond Syndrome was confirmed by low faecal elastase (<15 μg/g; normal >200 μg/g). For the remaining 4/11 cases diagnosed clinically as DBA, no pathogenic mutations were identified and MLPA excluded deletions in *RPL11*,* RPL35A*,* RPS17*,* RPS19*,* RPS26* and *RPL5*. Interestingly, the panel identified one additional patient with a previously reported *RPS19* mutation (Willig *et al*, 1999). This patient (Patient 44 [P44]), who had been submitted for investigation with a clinical diagnosis of CDA, presented at age 4 months with severe transfusion‐dependent anaemia, mild reticulocytosis (5·6%) and a normocellular marrow with relative erythroid hyperplasia. Two subsequent bone marrow aspirates at 7 and 12 months of age showed significant dyserythropoiesis, prompting Electron Microscopy (EM) examination which was reported as suspicious of CDA‐I, with ~50% of erythroblasts exhibiting ‘Swiss cheese heterochromatin’ (Fig 2 *,* Table 2).

#### Congenital dyserythropoietic anaemia

Nineteen cases were referred with probable CDA‐I and one with unclassified CDA (P38). Mutations in *CDAN1* were identified in 8/19 CDA‐I, including four cases of homozygosity for known (P39 and P40) or predicted (P51 and P53) pathogenic mutations, three cases of compound heterozygosity for one known and one predicted pathogenic mutation (P49, P50 and P55) and one compound heterozygosity for two known mutations (P56). The diagnosis of CDA‐I was confirmed by EM in all five patients with novel mutations in *CDAN1*. In P50 the 7 bp insertion results in a frameshift and premature stop codon (c.2971_2977dupGCAGCAG) predicted to result in transcripts susceptible to nonsense‐mediated decay. Sequencing of genomic DNA and complementary DNA (cDNA) confirmed the insertion was absent from cDNA rendering the patient functionally homozygous for the 2015C>T exon 14 mutation and therefore clinically affected (Fig S3). P38, referred with unclassified CDA, had compound heterozygosity for two pathogenic *KLF1* mutations previously reported in CDA‐IV, although not previously documented in the same patient (Gallienne *et al*, 2012; Viprakasit *et al*, 2014). The clinical features, early‐onset transfusion‐dependent, microcytic anaemia with reticulocytosis, jaundice, hepatosplenomegaly, high HbF (14·2%) and bone marrow erythroid hyperplasia with binucleate erythroblasts and chromatin bridges, were also consistent with CDA‐IV. Two further cases clinically diagnosed as CDA‐I (P44 and P54) were found to have mutations in non‐CDA genes (*RPS19* as discussed above) and *PKLR*. The latter (P54) was an 18‐month‐old transfusion‐dependent girl in whom bone marrow analysis showed erythroid hyperplasia with significant dyserythropoiesis and ~30% of erythroblasts were reported as exhibiting ‘Swiss‐cheese heterochromatin’, leading to a clinical diagnosis of CDA and pyruvate kinase (PK) levels were therefore not tested. After molecular analysis revealed compound heterozygosity for *PKLR* mutations, PK levels were tested and found to be low and central review of the marrow morphology was felt to be consistent with non‐specific dyserythropoiesis rather than CDA. The patient subsequently became transfusion independent after splenectomy (Fig 2 *,* Table 2).

#### Sideroblastic anaemia

Of three cases diagnosed by referring physicians as sideroblastic anaemia, one (P33) was homozygous for a mutation in *SLC25A38* predicted to be pathogenic due to a frameshift leading to an early termination codon consistent with the bone marrow findings. For the remaining two cases no pathogenic mutations were identified (Fig 2 *,* Table 2).

#### Enzyme deficiencies

Pathogenic mutations were identified in 1/4 cases with haemolytic anaemia suspected to be due to red cell enzyme deficiency. NP37 was a compound heterozygote for known pathogenic *PKLR* mutations and the diagnosis was confirmed with low PK levels. For the remaining three cases, no pathogenic mutations were identified (Fig 2 *,* Table 2).

#### Unexplained anaemia

Of the 19 patients where referring clinicians were unable to make any diagnosis using conventional investigations, ORCP produced a definitive molecular diagnosis in 2/19: PK deficiency (P9), and pyrimidine 5 nucleotidase (P5'N) deficiency (P14). P9, who has compound heterozygosity for two known pathogenic mutations in the *PKLR* gene, presented with neonatal liver failure and idiopathic inflammatory giant cell hepatitis and developed microcytic transfusion‐dependent anaemia with splenomegaly and reticulocytosis by 6 months of age. His blood film showed no basophilic stippling or echinocytes; PK assays were normal; and bone marrow examination showed only moderate erythroid hyperplasia; parental blood counts and films were normal. However, prompted by the targeted NGS results, repeat PK assays revealed reduced levels in the patient and both parents. P14 is a compound heterozygote for one known and one predicted pathogenic mutation in *NT5C3A*, the gene causing P5'N deficiency, confirmed by reduced enzyme levels. Interestingly, the blood film showed almost no basophilic stippling, explaining why this diagnosis was not suspected clinically. For the remaining 17/19 cases of unexplained anaemia, no pathogenic mutations in ORCP genes were identified although one case (P13) was found to have mutations of uncertain significance in *SEC23B,* which may be partly responsible for her anaemia (Table SIV) (Fig 2 *,* Table 2).

## Discussion

We set out to develop a clinical‐grade, targeted NGS panel to facilitate precise diagnosis of unexplained congenital anaemias, many of which had undergone repeated, unsuccessful investigations over many years. The use of targeted NGS in inherited anaemias/pancytopenia has been investigated by several groups in conditions such as DBA, bone marrow failure, Fanconi Anaemia and neonatal haemolytic anaemia (Gerrard *et al*, 2013; Collopy *et al*, 2014; De Rocco *et al*, 2014; Christensen *et al*, 2015; Ghemlas *et al*, 2015; Zhang *et al*, 2015). In contrast to these studies, our study employed novel analytical and validation strategies to ensure that the ORCP was suitable for reliable diagnosis in a clinical setting and is accredited by UKAS, the medical laboratory accreditation authority in the UK. Furthermore, we chose to take a relatively unbiased approach by including causative genes for conditions where anaemia may be part of a wider bone marrow failure syndrome as well as those for specific red cell disorders, such as DBA or CDA. Thus, our study contrasts with previously reported studies of targeted NGS, which have focused on well‐phenotyped cases, mostly with an established clinical diagnosis and a narrower range of genes. While this more targeted approach is likely to miss ‘atypical’ cases, we acknowledge that our broader approach probably led to a lower diagnostic yield in cases with classical features of unexplained anaemia without other associated cytopenias.

While the approach of choosing a narrow set of genes can speed up diagnosis and allow appropriate genetic counselling, it can, at best, confirm the clinically‐suspected diagnosis, as shown in previously published studies which reported >80% diagnostic yields (Gerrard *et al*, 2013; Collopy *et al*, 2014; De Rocco *et al*, 2014; Christensen *et al*, 2015; Ghemlas *et al*, 2015; Zhang *et al*, 2015). Similarly, for most of our cases, confirmation of a suspected diagnosis by molecular analysis also allowed provision of accurate information for genetic counselling for the first time in 17/57 (~30%) of cases. Particularly crucial were the seven patients in whom we established the molecular basis of DBA and accurate genetic counselling was provided by identifying *de novo* cases, where the recurrence risk is extremely low, from familial cases. Furthermore, appropriate treatment, surveillance and prophylaxis were also instituted for the first time for P14 (P5'N deficiency), P23 (SDS) and P44 (DBA).

Use of the ORCP to make an accurate molecular diagnosis had life‐changing impacts for several of the patients and their families, particularly for patients P9 and P54 for whom a new diagnosis of PK deficiency led to transfusion‐independence and cessation of iron chelation following splenectomy. PK deficiency had previously been missed due to repeatedly normal PK enzyme levels, a well‐recognized pitfall especially in transfusion‐dependent patients (Koralkova *et al*, 2014). For P44, initially misdiagnosed as CDA‐I, the correct diagnosis of DBA dramatically changed the prognosis, response to therapy and genetic counselling offered to the family. This case was atypical for DBA, as there was initial reticulocytosis and erythroid hyperplasia on three separate marrow aspirations, so DBA was not considered in the differential diagnosis.

Targeted NGS offers several advantages over the traditional specialized investigations used to diagnose congenital anaemia. Firstly, the risk of a wrong diagnosis is reduced given the practical and technical limitations of biochemical assays and EM interpretation without molecular confirmation. Indeed, our study shows that mis‐diagnosis of CDA‐I is fairly common, as the degree of bone marrow dyserythropoiesis in haemolytic conditions, although noted in previous case reports (Haija *et al*, 2014), remains under‐appreciated. Cases of CDA‐I submitted for molecular analysis were not felt to show CDA‐I‐ specific features when reviewed by our specialist pathologist, highlighting the difficulty of relying on morphological findings alone to make a diagnosis of CDA. A second advantage of targeted NGS is that, unlike most tests for unexplained congenital anaemia, it can be reliably performed in regularly‐transfused patients and samples can be sent by post without specialized processing or shipping.

The validation strategy we have used fulfilled the requirements set out by the ACMG for NGS panels to be used in the diagnostic setting, in contrast to previous studies (Gerrard *et al*, 2013; Collopy *et al*, 2014; De Rocco *et al*, 2014; Christensen *et al*, 2015; Ghemlas *et al*, 2015; Zhang *et al*, 2015). Crucially, assessment of coverage in these studies relied on assessment of overall coverage and did not account for the coverage at the specific locations where mutations have previously been described in all of the genes tested. We therefore developed a novel bioinformatic algorithm, CoverMi, to assess the per‐base read coverage of all locations where mutations have previously been described. This approach, which has not previously been reported, allows haematologists to make the distinction between the absence of mutations and the lack of adequate sequencing reads at the locations of known mutations. We propose that such an approach should be essential for a NGS panel to be used for clinical diagnosis and here we make it freely available to other users.

Typically, diagnosis of unexplained congenital anaemia involves sequential investigations including routine haematological and biochemical tests followed by specialized tests (e.g. red cell enzyme assays, bone marrow morphology) and, finally, testing of individual genes directed by these investigations. Our targeted panel may allow the diagnostic pathway to be shortened by performing NGS before specialized investigations. For many patients this would dramatically reduce the time to diagnosis and the number of expensive, complex tests, as specific confirmatory tests can be targeted to individual patients to confirm the molecular diagnosis. Indeed, the average time to diagnosis for the five new/changed diagnoses in our study was 3·3 years, rather than 3 months had NGS been available earlier. However, we acknowledge that several strategies will be needed to increase the diagnostic yield of targeted NGS for congenital anaemia before this approach will replace conventional diagnostic approaches. In particular, we plan to include a larger number of red cell enzyme genes and add red cell membrane genes to the panel. Furthermore, the diagnostic utility of such panels depends on a regular process of review to accommodate relevant newly identified pathogenic and modifying mutations in genes and their promoters and regulatory elements (Crispino & Weiss, 2014). It is likely that whole exome sequencing or whole genome sequencing, which provide a much more comprehensive, unbiased approach, will replace targeted sequencing in the future. At present, however, the use of a restricted, targeted panel requires less complex analysis with fewer incidental findings (Sun *et al*, 2015) and is a practical approach to achieving a rapid, accurate diagnosis where routine investigations have failed.

In conclusion, we demonstrate the feasibility of using a clinical‐grade NGS panel in routine clinical diagnosis of rare congenital anaemias. This approach has a good diagnostic yield (38·6%), which leads to improved, tailored patient management. Our NGS data validation shows 100% specificity and reproducibility with high sensitivity. Finally, evidence from other inherited diseases indicates that accurate and timely diagnosis improves patient well‐being by reducing the anxiety associated with inaccurate prognostic information (Lenhard *et al*, 2005).

## Author contributions

NBAR, EAW, KW, SO, HD, DF, JM and MP performed experiments; NBAR, EAW, CB, AM, CH, PC, J Cullis, J Curtin, BG, GH, MM, AQ, JSG, NS, JT and UT analysed results and made the figures; NBAR, SH, CB, WA, CH, PC, DR, DH, AS and IR designed the research and wrote the paper.

## Conflicts of interest

The authors declare that they have no conflicts of interest.

## Supporting information


**Fig S1.** Diagram of the work_ow from receipt of sample to production of a clinical report.

**Fig S2.** Schematic diagram of an amplicon with uneven distribution of forward and reverse reads.

**Fig S3.** Sanger sequencing analysis of novel frameshift mutation in *CDAN1*.

**Table SI.** Clinical details of all 57 samples submitted for analysis on the Oxford Red Cell Panel.
**Table SII.** Nomenclature and references for the 33 genes on the Oxford Red Cell Panel.
**Table SIII.** Details of all 32 mutations identified in samples from the 57 patients analysed on the ORCP.
**Table SIV.** Mutations of uncertain significance identified in samples from 3 patients analysed on the ORCP
