# Supplementary material for: A novel 33‐Gene targeted resequencing panel provides accurate, clinical‐grade diagnosis and improves patient management for rare inherited anaemias
Source: Br J Haematol. 2016 Jul 19;175(2):318–30. doi: 10.1111/bjh.14221 (PMC5132128; doi:10.1111/bjh.14221)

Supplementary Figure 1.
Diagram of the workflow from receipt of sample to production of a clinical report.

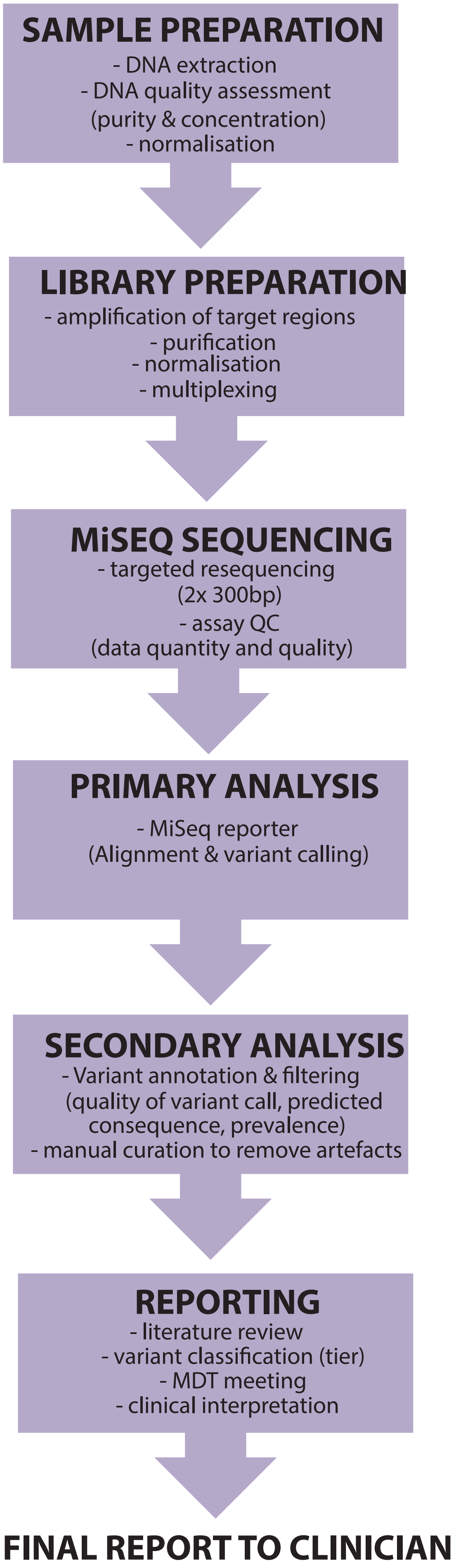

Supplement: Supplementary file 1 — Fig S1. Diagram of the work_ow from receipt of sample to production of a clinical report. [file BJH-175-318-s001.pdf]
