# Supplementary material for: A novel 33‐Gene targeted resequencing panel provides accurate, clinical‐grade diagnosis and improves patient management for rare inherited anaemias
Source: Br J Haematol. 2016 Jul 19;175(2):318–30. doi: 10.1111/bjh.14221 (PMC5132128; doi:10.1111/bjh.14221)

Supplementary Fig 2: Schematic diagram of an amplicon with uneven distribution of forward and reverse reads. There is uneven coverage on the forward (65) and reverse (3) reads. Primers for all amplicons are designed with an overlapping region between the forward and reverse reads and current commercially available software calculates amplicon coverage as the average number of reads across the amplicon (here the average amplicon coverage is 34 reads, based on 65 forward reads and 3 reverse reads). Also shown is the site of 2 previously described mutations (A and B) a clinical-grade NGS panel needs to be able to detect in order to determine whether or not a patient has a mutation present at either of those sites. Based on the average coverage across the amplicon, it would be assumed that both mutations would be detected if present. However, actual coverage at base-pair level reveals that only mutation A would be discoverable as the coverage is good (65 reads) whereas mutation B, with only 3 reads, would fall below the lower limit cut-off (>30 reads) and therefore would not be detected. Accurate read coverage data at each individual base location is therefore necessary to differentiate between the absence of mutations in regions with adequate coverage from the absence of mutations due to insufficient data.

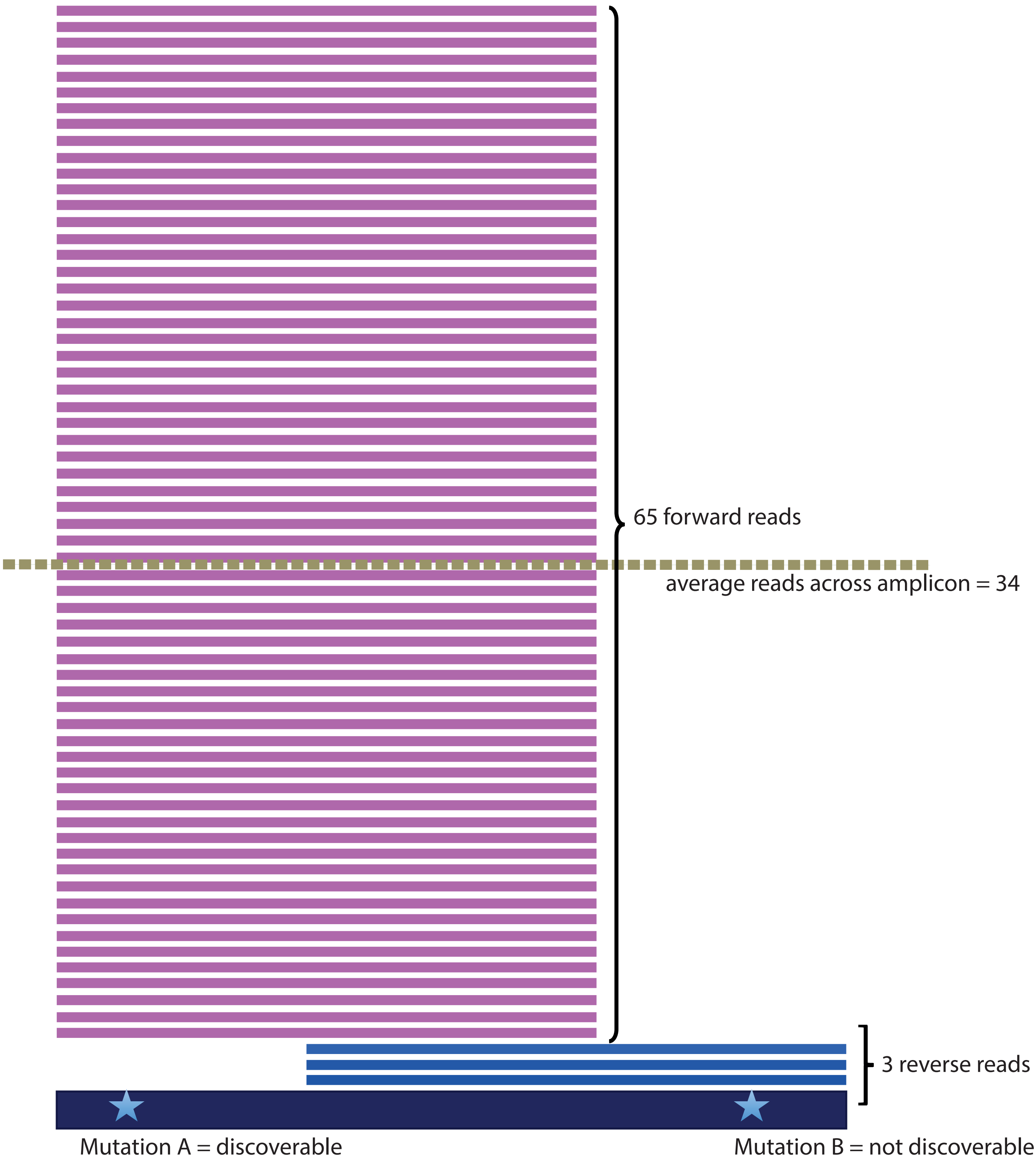

Supplement: Supplementary file 2 — Fig S2. Schematic diagram of an amplicon with uneven distribution of forward and reverse reads. [file BJH-175-318-s002.pdf]
