# Supplementary material for: A novel 33‐Gene targeted resequencing panel provides accurate, clinical‐grade diagnosis and improves patient management for rare inherited anaemias
Source: Br J Haematol. 2016 Jul 19;175(2):318–30. doi: 10.1111/bjh.14221 (PMC5132128; doi:10.1111/bjh.14221)

Supplementary Fig 3. Sanger sequencing analysis of novel frameshift mutation in *CDAN1*.
(A) Sanger sequencing of complementary DNA (cDNA) and genomic DNA (gDNA) of from cultured patient lymphocytes for patient 50 for exon 23 of CDAN1. Heterozygosity for a 7bp insertion in gDNA of CDAN1 causing a frameshift (orange brackets) while cDNA, which shows only the expressed allele, is wild type in this region.
(B) Diagram illustrating nonsense mediated decay of the frameshifted allele and the resultant functional homozygosity.

A

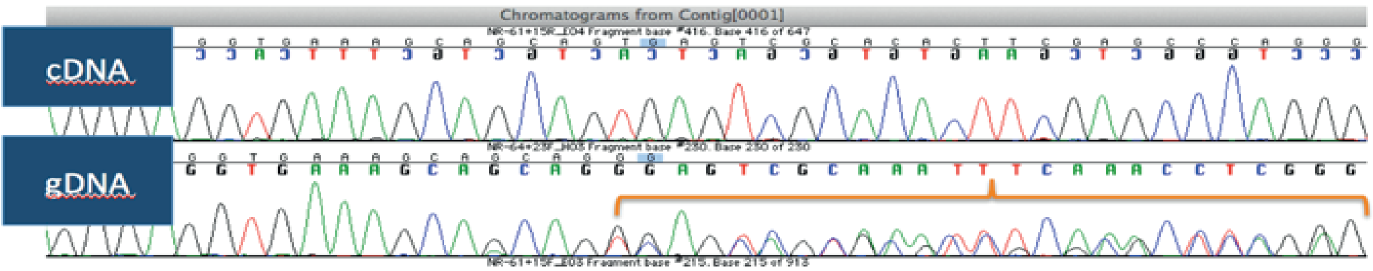

B

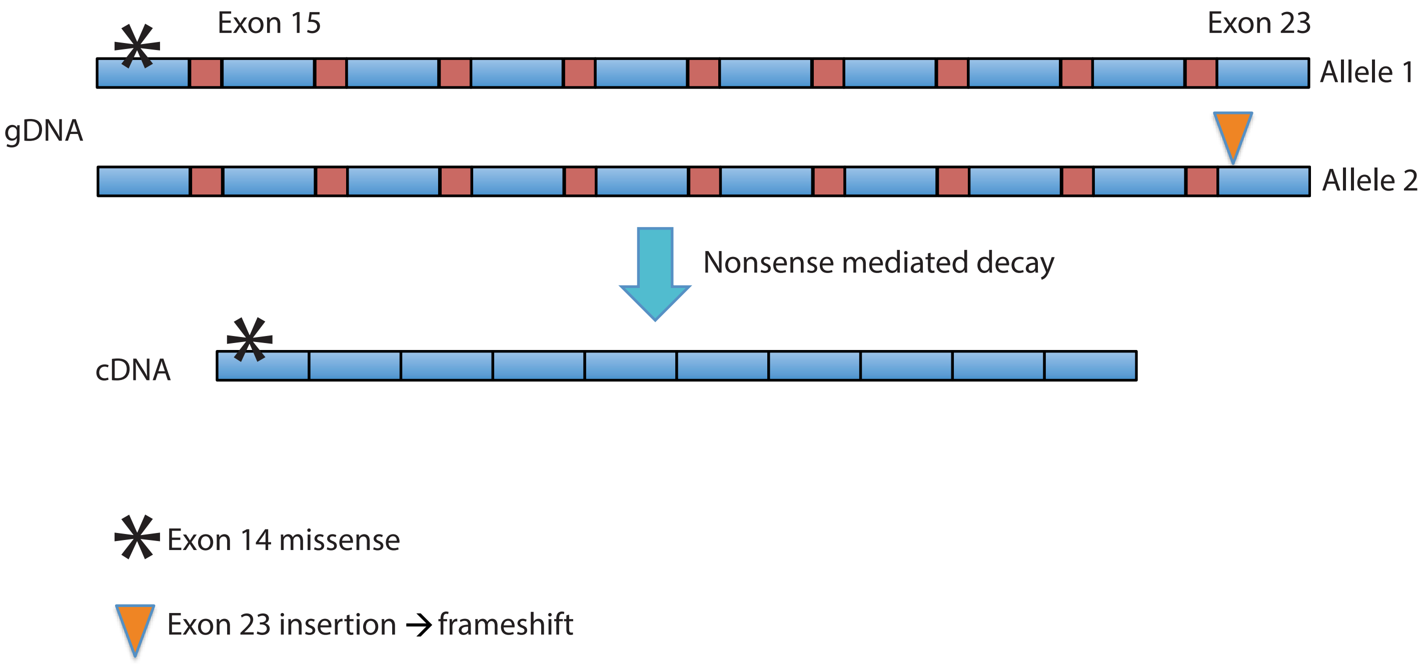

Supplement: Supplementary file 3 — Fig S3. Sanger sequencing analysis of novel frameshift mutation in CDAN1. [file BJH-175-318-s003.pdf]
