# Supplementary material for: A novel 33‐Gene targeted resequencing panel provides accurate, clinical‐grade diagnosis and improves patient management for rare inherited anaemias
Source: Br J Haematol. 2016 Jul 19;175(2):318–30. doi: 10.1111/bjh.14221 (PMC5132128; doi:10.1111/bjh.14221)
Supplement: Supplementary file 4 — Table SI. Clinical details of all 57 samples submitted for analysis on the Oxford Red Cell Panel. Table SII. Nomenclature and references for the 33 genes on the Oxford Red Cell Panel. Table SIII. Details of all 32 mutations identified in samples from the 57 patients analysed on the ORCP. Table SIV. Mutations of uncertain significance identified in samples from 3 patients analysed on the ORCP [file BJH-175-318-s004.docx]

**Supplementary Table I:** Clinical details of all 57 samples submitted for analysis on the Oxford Red Cell Panel

| **Patient ID** | **Working diagnosis** | **Ethnic origin** | **Age at presentation** | **Lineages affected** | **Transfusion dependence** | **Diagnosis after NGS** | **Comments** |
| --- | --- | --- | --- | --- | --- | --- | --- |
| Patient 1 (P1) | Unexplained anaemia | Caucasian | Neonate | Pancytopenia | Yes | Unexplained anaemia | BM: erythroid hyperplasia and dysplasia, reduced myelopoiesis, dysplastic megakaryocytes |
| Patient 2 (P2) | Unexplained anaemia | Middle Eastern | 5 years | Anaemia only | No | Unexplained anaemia | Normal HPLC  Family history of unexplained anaemia |
| Patient 3 (P3) | Unexplained anaemia | Middle Eastern | 9 months | Anaemia only | No | Unexplained anaemia | Normal HPLC |
| Patient 4 (P4) | Unexplained anaemia | Middle Eastern | 6 years | Anaemia only | No | Unexplained anaemia | Carrier for IVS1-5(G>C) which is usually mild, hypoplastic marrow. Anaemia severe (Hb 61g/L, MCV 65fl) |
| Patient 5 (P5) | Unexplained anaemia | Caucasian | 6 years | Anaemia only | No | Unexplained anaemia | Transient mild neutropenia |
| Patient 6 (P6) | Unexplained anaemia | Caucasian | 5 months | Pancytopenia | Yes from 5 months | Unexplained anaemia | Neonatal anaemia, frequent infections |
| Patient 7 (P7) | Unexplained anaemia | Caucasian | 8 years | Anaemia only | No | Unexplained anaemia | Macrocytosis (MCV 125fl) despite α thalassaemia trait, splenectomy |
| Patient 8 (P8) | Unexplained anaemia | Caucasian | 7 years | Anaemia only | Yes from 7 | Unexplained anaemia | Recurrent infections but normal white cell count, developmental delay |
| Patient 9 (P9) | Unexplained  anaemia | Caucasian | 6 months | Anaemia only | Yes | PK deficiency | Hepatic failure (“giant cell hepatitis” on biopsy) as neonate, resolved. Normal reticulocytes, normal PK level, normal blood smear, mild splenomegaly. |
| Patient 10 (P10) | Unexplained anaemia | Afro-Carribean | 1 year | Anaemia only | Yes | Unexplained anaemia | Diagnosis of SIFD (syndrome of sideroblastic anaemia, immune deficiency, fever and developmental delay) made after *TRNT1* mutations identified. |
| Patient 11 (P11) | Unexplained anaemia | Caucasian | 1 year | Anaemia only | Yes from birth/utero (two IUTs) | Unexplained anaemia | Erythroid hyperplasia on marrow, no sideroblastic anaemia, no CDA. |
| Patient 12 (P12) | Unexplained anaemia | Asian | 18 years | Anaemia only, then pancytopenia | Yes after 1 year | Unexplained anaemia | Initial BM: reduced erythropoiesis, repeat after 1 year confirmed evolution to hypoplastic MDS/AA |
| Patient 13 (P13) | Unexplained anaemia | Caucasian | In utero | Anaemia only | Yes | CDA-2 | Required 2x IUTs then none needed until 1 year old. VSD and ventricular hypertrophy. BMA done after molecular diagnosis is consistent with CDA-2 (dyserythropoiesis and binucleate cells) but EM inconclusive. |
| Patient 14 (P14) | Unexplained anaemia | Afro-Carribean | 7 years | Anaemia only | No | Pyrimidine-5'-nucleotidase deficiency | Mild reticulocytosis |
| Patient 15 (P15) | Unexplained anaemia | Caucasian | 1 year | Pancytopenia | Yes | Unexplained anaemia | Frequent infections. Mother had HSCT for ?MDS but no known MDS-associated mutations. |
| Patient 16 (P16) | Unexplained anaemia | Caucasian | 1 year | Anaemia only | Yes | Unexplained anaemia | Hb 72 g/L, HbF 4.7%, developed liver failure |
| Patient 17 (P17) | Unexplained anaemia | Caucasian | 4 years | Anaemia only | No | Unexplained anaemia | Fetal anaemia, poor growth, microcephaly |
| Patient 18 (P18) | Unexplained anaemia | Caucasian | 4 years | Pancytopenia | Yes | Unexplained anaemia | Patient died of infection age 5 years |
| Patient 19 (P19) | Unexplained anaemia | Asian | 16 years | Anaemia only | No | Unexplained anaemia | Haemoglobinuria |
| Patient 20 (P20)  G155793T | Diamond-Blackfan anaemia | Caucasian | 2 years | Anaemia only | Yes | Unexplained anaemia | 7-8 weekly transfusions |
| Patient 21 (P21) | Diamond-Blackfan anaemia | Caucasian | 5 months | Anaemia only | Yes | Diamond-Blackfan anaemia | Failure to thrive, poor weight gain, lethargy, severe anaemia |
| Patient 22 (P22) | Diamond-Blackfan anaemia | Caucasian | 6 months | Anaemia only | Yes | Diamond-Blackfan anaemia | Deafness, short stature, broad nasal bridge, hypertelorism, low set ears, recurrent ear infections, unresponsive to steroids |
| Patient 23 (P23) | Diamond-Blackfan anaemia | Caucasian | 16 months | Anaemia only | No | Diamond-Blackfan anaemia | Intrauterine growth restriction (2^nd^ centile, parents both 98^th^ centile) |
| Patient 24 (P24) | Diamond-Blackfan anaemia | Caucasian | 12 years | Anaemia only | Yes | Unexplained anaemia | BM erythroid hypoplasia, steroid responsive |
| Patient 25 (P25) | Diamond-Blackfan anaemia | Middle Eastern | 9 years | Anaemia only | No | Diamond-Blackfan anaemia | BM erythroid hypoplasia, steroid responsive high eADA |
| Patient 26 (P26) | Diamond-Blackfan anaemia | Maori | 11 years | Anaemia only | No | Diamond-Blackfan anaemia | BM erythroid hypoplasia, steroid responsive high eADA |
| Patient 27 (P27) | Diamond-Blackfan anaemia | Caucasian | 18 months | Anaemia only | Yes | Unexplained anaemia | Responsive to steroids |
| Patient 28 (P28) | Diamond-Blackfan anaemia | Caucasian | 3 years | Anaemia only | Yes | Unexplained anaemia | Micrognathia |
| Patient 29 (P29) | Diamond-Blackfan anaemia | Caucasian | 2 years | Anaemia only | Yes | Unexplained anaemia | Reticulocytopenia |
| Patient 30 (P30) | Diamond-Blackfan anaemia | Caucasian | 3 years | Anaemia only | Yes | Diamond-Blackfan anaemia | Hypocellular bone marrow, cleft palate, Pierre Robin syndrome |
| Patient 31 (P31) | Sideroblastic anaemia | Asian | 15 years | Anaemia only | No | Unexplained anaemia | BM: ring sideroblasts |
| Patient 32 (P32) | Sideroblastic anaemia | Middle Eastern | 2 years | Anaemia only | No | Unexplained anaemia | BM: ring sideroblasts Consanguinous |
| Patient 33 (P33) | Sideroblastic anaemia | Asian | 3 months | Anaemia only | Yes | Sideroblastic anaemia | BM: ring sideroblasts  Unresponsive to pyridoxine  Microcytosis |
| Patient 34 (P34) | Enzyme deficiency | Caucasian | 8 years | Anaemia only | Yes | Unexplained anaemia | Normal G6PD and PK levels |
| Patient 35 (P35) | Enzyme deficiency | Caucasian | 58 years | Anaemia only | No | Unexplained anaemia | Haemolytic anaemia |
| Patient 36 (P36) | Enzyme deficiency | Caucasian | 64 years | Anaemia only | No | Unexplained anaemia | Haemolytic anaemia |
| Patient 37 (P37) | Enzyme deficiency | Caucasian | 1yr 9 mo | Anaemia only | No | PK deficiency | Haemolytic anaemia |
| Patient 38 (P38) | Congenital Dyserythropoietic Anaemia | Asian | 5 months | Anaemia only | Yes from birth | Unexplained anaemia | HbF 14.2%, HbA 78.1%, HbA2 7.4%. BM: erythroid hyperplasia, binucleate cells and chromatin bridges |
| Patient 39 (P39) | Congenital Dyserythropoietic Anaemia | Caucasian | Neonate | Anaemia only | No | CDA-I | Neonatal anaemia and prolonged jaundice, BMA consistent with CDA-I. Consanguinity |
| Patient 40 (P40) | Congenital Dyserythropoietic Anaemia | Middle Eastern | 3 years | Anaemia only | No | CDA-I | BM erythroid hyperplasia, significant dyserythropoiesis  Consanguinity; affected sibling |
| Patient 41 (P41) | Congenital Dyserythropoietic Anaemia | Caucasian | 19 years | Anaemia only | No | Unexplained anaemia | Neonatal anaemia, splenomegaly |
| Patient 42 (P42) | Congenital Dyserythropoietic Anaemia | Asian | 14 years | Anaemia only | No | Unexplained anaemia | Failure to thrive, facial dysmorphism, limb dysplasia, BM dyserythropoiesis |
| Patient 43 (P43) | Congenital Dyserythropoietic Anaemia | Caucasian | 58 years | Anaemia only | No | Unexplained anaemia | Family history of anaemia  Splenomegaly |
| Patient 44 (P44) | Congenital Dyserythropoietic Anaemia | Afro-Carribean | 4 months | Anaemia only | Yes | Diamond-Blackfan Anaemia | Reticulocyte count initially normal. Dyserythropoiesis noted on BM. "Swiss cheese heterochromatin" on EM. Single dysplastic kidney |
| Patient 45 (P45) | Congenital Dyserythropoietic Anaemia | Chinese | 47 years | Anaemia only | No | Unexplained anaemia | MCV 90fl, reticulocytopenia |
| Patient 46 (P46) | Congenital Dyserythropoietic Anaemia | Caucasian | 27 years | Anaemia only | No | Unexplained anaemia | BM: erythroid dysplasia |
| Patient 47 (P47) | Congenital Dyserythropoietic Anaemia | Caucasian | 3 years | Anaemia only | No | Unexplained anaemia | BM: marked erythroid hyperplasia, mild dyserythropoieis, developmental delay |
| Patient 48 (P48) | Congenital Dyserythropoietic Anaemia | Caucasian | 5 years | Anaemia only | No | Unexplained anaemia | BM: normocellular, mild dyserythropoiesis, neonatal anaemia, facial dysmorphism |
| Patient 49 (P49) | Congenital Dyserythropoietic Anaemia | Caucasian | Childhood | Anaemia only | No | CDA-I | BM EM typical for CDA-I |
| Patient 50 (P50) | Congenital Dyserythropoietic Anaemia | Caucasian | 4 years | Anaemia only | No | CDA-I | BM EM typical for CDA-I, Iron overload. Angioid streak.  Brother affected |
| Patient 51 (P51) | Congenital Dyserythropoietic Anaemia | Caucasian | 31 years | Anaemia only | No | CDA-I | Neonatal anaemia and prolonged jaundice. Re-presented with fatigue at 31 yrs, BM EM typical for CDA-I. Iron overload. Sister affected |
| Patient 52 (P52) | Congenital Dyserythropoietic Anaemia | Middle Eastern | Childhood | Anaemia only | Yes | Unexplained anaemia | Splenomegaly |
| Patient 53 (P53) | Congenital Dyserythropoietic Anaemia | Caucasian | 5 years | Anaemia only | No | CDA-I | BM: EM suggestive but not diagnostic of CDA-I, retinoblastoma, synophrys, high palate, limited extension elbows, neonatal prolonged jaundice |
| Patient 54 (P54) | Congenital Dyserythropoietic Anaemia | Caucasian | 18 months | Anaemia only | Yes until splenectomy | PK deficiency | Haemolysis with reticulocytosis but BM suggestive of CDA and EM reported as CDA-I |
| Patient 55 (P55) | Congenital Dyserythropoietic Anaemia | Caucasian | 20 years | Anaemia only | No | CDA-I | BM: light microscopy suggestive of CDA-I |
| Patient 56 (P56) | Congenital Dyserythropoietic Anaemia | Caucasian | Childhood | Anaemia only | No | Unexplained anaemia | CDA-I on EM |
| Patient 57 (P57) | Congenital Dyserythropoietic Anaemia | Caucasian | Neonate | Anaemia only | Yes until splenectomy | Unexplained anaemia | CDA-I on EM, extramedullary haematolopoiesis |

*:Working diagnosis made by the referring clinician

Key: CDA: congenital dyserythropoietic anaemia, PK: pyruvate kinase, BM: bone marrow, EM: electron microscopy, HPLC: high performance liquid chromatography, MCV: mean cell volume, Hb: haemoglobin, G6PD: glucose-6-phosphate dehydrogenase, eADA: erythrocyte adenine deaminase, MDS: myelodysplastic syndrome, HSCT: haematopoietic stem cell transplant, IUT: intra-uterine transfusion, NGS: next-generation sequencing

**Supplementary Table II: Nomenclature and references for the 33 genes on the Oxford Red Cell Panel.**

| **HGNC standard name and symbol of the gene** | **HGNC**  **number** | **OMIM**  **number** | **OMIM standard name of condition and symbol** | **Mode of inheritance** | **Evidence of association between gene(s) and condition** | **% of horizontal coverage of gene** |
| --- | --- | --- | --- | --- | --- | --- |
| codanin 1  *CDAN1* | HGNC:1713 | 607465 | Anaemia, Congenital Dyserythropoietic, Type I | AR | Dgany O (2002). Am J Hum Genet 71(6):1467-74 | 94.4 |
| Sec23 homolog B (S. cerevisiae)  *SEC23B* | HGNC:10702 | 610512 | Anaemia, Congenital Dyserythropoietic, Type II | AR | Bianchi P (2009). Hum Mutat 30(9):1292-8. | 100 |
| chromosome 15 open reading frame 41  *C15orf41* | HGNC:26929 | N/A | N/A | AR | Babbs C (2013). Haematologica 98(9):1383-7. | 100 |
| GATA binding protein 1 (globin transcription factor 1) *GATA1* | HGNC:4170 | 305371 | Anaemia, X-linked, with/without neutropenia and/or platelet abnormalities | X-linked | Nichols KE (2000) Nat Genet 24(3):266-70 | 100 |
| *KLF1*  Kruppel-like factor 1 (erythroid) | HGNC:6345 | 600599 | Anaemia, Dyserythropoietic congenital, type IV | AD | Arnaud L (2010) Am J Hum Genet 87(5):721-7. | 100 |
| *KIF23*  Kinesin Family Member 23 | HGNC:6392 | 605064 | Anaemia, Congenital Dyserythropoietic, Type III | AD | Liljeholm M (2013) Blood 121(23):4791-9. | 100 |
| *ALAS2*  aminolevulinate, delta-, synthase 2 | HGNC:397 | 301300 | Anaemia, Hereditary Sideroblastic X-Linked | X-linked | Cotter PD (1995) J Clin Invest 96(4):2090-6 | 100 |
| *SLC25A38*  solute carrier family 25, member 38 | HGNC:26054 | 610819 | Anaemia, Sideroblastic, Pyridoxine-Refractory, Autosomal Recessive | AR | Guernsey DL (2009) Nat Genet 41(6):651-3. | 100 |
| *DKC1*  dyskeratosis congenita 1, dyskerin | HGNC:2890 | 300126 | Dyskeratosis Congenita, X-Linked | X-linked | Knight SW (1999) Br J Haematol 107(2):335-9. | 100 |
| *NHP2*  NHP2 ribonucleoprotein | HGNC:14377 | 606470 | Dyskeratosis Congenita, Autosomal Recessive, 2 | AR | Vulliamy T (2008) Proc Natl Acad Sci 105(23):8073-8. | 100 |
| *NOP10*  NOP10 ribonucleoprotein | HGNC:14378 | 606471 | Dyskeratosis Congenita, Autosomal Recessive, 1 | AR | Walne AJ (2007) Hum Mol Genet 16(13):1619-29. | 83.3 |
| *NT5C3A*  5'-nucleotidase, cytosolic IIIA | HGNC:17820 | 606224 | Haemolytic Anaemia Due To Umph1 Deficiency | AR | Marinaki AM (2001) Blood 97(11):3327-32 | 100 |
| *TERC*  telomerase RNA component | HGNC:11727 | 602322 | Dyskeratosis Congenita, Autosomal Dominant, 1 | AD | Vulliamy T (2004) Nat Genet 36(5):447-9. | 100 |
| *TERT*  telomerase reverse transcriptase | HGNC:11730 | 187270 | Bone marrow failure, telomere-related, 1 | AD  AR | Vulliamy T (2005) Blood Cells Mol Dis 34(3):257-63. | 100 |
| *TINF2*  TERF1 (TRF1)-interacting nuclear factor 2 | HGNC:11824 | 604319 | Dyskeratosis Congenita, Autosomal Dominant, 3 | AD | Savage SA (2008) Am J Hum Genet 82(2):501-9 | 93 |
| *SBDS*  Shwachman-Bodian-Diamond syndrome | HGNC:19440 | 607444 | Shwachman-Diamond Syndrome | AR | Boocock GR (2003) Nat Genet 33(1):97-101 | 100 |
| *G6PD*  glucose-6-phosphate dehydrogenase | HGNC:4057 | 305900 | Haemolytic anaemia due to G6PD deficiency | X-linked | HGMD ® website: http://www.biobase-international.com/product/hgmd | 100 |
| *PKLR*  pyruvate kinase, liver and RBC | HGNC:9020 | 609712 | Pyruvate Kinase Deficiency | AR | HGMD ® website: http://www.biobase-international.com/product/hgmd | 97.6 |
| *RPL11*  ribosomal protein L11 | HGNC:10301 | 604175 | Diamond-Blackfan Anaemia 7 | AD | Gazda HT (2008) Am J Hum Genet 83(6):769-80 | 100 |
| *RPL26*  ribosomal protein L26 | HGNC:10327 | 603704 | Diamond-Blackfan Anaemia 11 | AD | Gazda HT (2012) Hum Mutat 33(7):1037-44 | 100 |
| *RPL35A*  ribosomal protein L35a | HGNC:10345 | 180468 | Diamond-Blackfan Anaemia 5 | AD | Farrar JE (2008) Blood 112(5):1582-92 | 100 |
| *RPL5*  ribosomal protein L5 | HGNC:10360 | 603634 | Diamond-Blackfan Anaemia 6 | AD | Gazda HT (2008) Am J Hum Genet 83(6):769-80 | 100 |
| *RPS10*  ribosomal protein S10 | HGNC:10383 | 603632 | Diamond-Blackfan Anaemia 9 | AD | Doherty L (2010) Am J Hum Genet 86(2):222-8 | 100 |
| *RPS17*  ribosomal protein S17 | HGNC:10397 | 180472 | Diamond-Blackfan Anaemia 4 | AD | Cmejla R (2007) Hum Mutat 28(12):1178-82 | 100 |
| *RPS19*  ribosomal protein S19 | HGNC:10402 | 603474 | Diamond-Blackfan Anaemia 1 | AD | Draptchinskaia N (1999) Nat Genet 21(2):169-75. | 100 |
| *RPS24*  ribosomal protein S24 | HGNC:10411 | 602412 | Diamond-Blackfan Anaemia 3 | AD | Gazda HT (2006) Am J Hum Genet 79(6):1110-8 | 94.3 |
| *RPS7*  ribosomal protein S7 | HGNC:10440 | 603658 | Diamond-Blackfan Anaemia 8 | AD | Gazda HT (2008) Am J Hum Genet 83(6):769-80 | 100 |
| *RPL19*  ribosomal protein L19 | HGNC:10312 | 180466 | Diamond-Blackfan Anaemia 12 | AD | Gerrard G (2013) Br J Haematol 162(4):530-6 | 95.4 |
| *RPL27*  ribosomal protein L27 | HGNC: 10328 | 607526 | Diamond-Blackfan Anaemia | AD | Wang R (2015) Br J Haematol 168(6):854-64 | 84.1 |
| *RPL9*  ribosomal protein L9 | HGNC: 10369 | 603686 | Diamond-Blackfan Anaemia | AD | Gerrard G (2013) Br J Haematol 162(4):530-6 | 100 |
| *RPS26*  ribosomal protein S26 | HGNC: 10414 | 603701 | Diamond-Blackfan Anaemia 10 | AD | Doherty L (2010) Am J Hum Genet 86(2):222-8 | 100 |
| *RPS27*  ribosomal protein S27 | HGNC: 10416 | 603702 | Diamond-Blackfan Anaemia | AD | Gerrard G (2013) Br J Haematol 162(4):530-6 | 97 |
| *RPS29*  ribosomal protein S29 | HGNC: 10419 | 603633 | Diamond-Blackfan Anaemia | AD | Gerrard G (2013) Br J Haematol 162(4):530-6 | 100 |

The HUGO gene nomenclature number (HGNC), name and gene symbol is given for each gene, as well as the OMIM (Online

Mendelian Inheritance in Man) number and disease name and symbol. The mode of inheritance of each disorder associated

with each gene is given, along with key publications which established the pathogenicity of each gene. In the last column,

the overall coverage of each gene is given, as determined in the design file of the panel.

Key: AR: autosomal recessive; AD: autosomal dominant

**Supplementary Table III:** Details of all 32 mutations identified in samples from the 57 patients analysed on the ORCP

| **ID** | **Gene** | **Location** | **Variant** | **Depth** | **cDNA** | **Protein** | **Reference** | **SIFT** | **Polyphen2** | **EVS** | Tier |
| --- | --- | --- | --- | --- | --- | --- | --- | --- | --- | --- | --- |
| Patient 9  (P9) | *PKLR* | 1:  155261636 | C>T | 611/1278  (48.2%) | c.1529G>A | p.Arg510Gln | Baronciani  PNAS 1993 | DEL  (0.01) | PROB DAM  (0.994) | TT=0/  TC=9/  CC=6494 | 1 |
|  |  | 1:  155264517 | C>A | 730/1486  (49.4%) | c.721G>T | p.Glu241Ter |  | N/A | N/A | No | 2 |
| Patient 14  (P14) | *NT5C3A* | 7:  33057113 | G>G/A | 56/128  (52%) | c.646C>T | p.Gln216Ter | Marinaki  Blood 2001 |  |  | No | 1 |
|  |  | 7:  33059337 | C>C/T | 163/420  (38.8%) | c.456-1G>A |  |  |  |  | No | 2 |
| Patient 20  (P20) | *RPL5* | 1:  93299216 | AG>A | 519/1044  (49.7%) | c.189delG | p.Ile64Leu  fsTer6 |  |  |  | TT=0/  TA=1/  AA=6502 | 2 |
| Patient 21  (P21) | *RPS19* | 19:  42373769 | CG>CG/C | 2171/5000  (43.4%) | c.358delG | p.Gly120Ala  fsTer4 |  |  |  | TT=0/  TC=1/  CC=6502 | 2 |
| Patient 22  (P22) | *RPL5* | 1:  93301807 | G>T | 625/1261  (49.6%) | c.385G>T | p.Glu129Ter |  |  |  | No | 2 |
| Patient 23  (P23) | *SBDS* | 7:  66459197 | A>G | 305/313  (99.3%) | c.258+2T> |  | Boocock  2003 |  |  | GG=0/  GA=37/  AA=6466 | 1 |
| Patient 25  (P24) | *RPS26* | 12:  56436388 | TA>T | 293/755  (38.8%) | c.181+3delA |  |  |  |  | No | 3 |
| Patient 26  (P26) | *RPL11* | 1:  24020302 | T>A | 40/100  (40%) | c.163_164insA | p.Tyr55Ter |  |  |  | No | 2 |
| Patient 30  (P30) | *RPL5* | 1:  93299199 | CAG>C | 527/1101  (47.9%) | c.172_173delAG | p.Asp59Tyr  fsTer53 |  |  |  | No | 2 |
| Patient 33  (P33) | *SLC25A38* | 3:  39433366 | G>GT | 160/162  (98.8%) | c.479_480insT | p.Ile161Tyr  fsTer12 |  |  |  | No | 2 |
| Patient 37  (P37) | *PKLR* | 1:  155261636 | C>T | 548/1161  (47.3%) | c.1529G>A | p.Arg510Gln | Baronciani  1993 | DEL  (0.01) | PROB DAM  (0.994) | TT=0/  TC=8/  CC=4292 | 1 |
|  |  | 1:  155264118 | T>A | 1073/2190  (49.1%) | c.1024A>T | p.Ile342Phe | Barionciani  1998 | DEL  (0) | PROB DAM  (0.998) | No | 1 |
| Patient 38  (P38) | *KLF1* | 19:  12995785 | C>T | 235/449  (52.8%) | c.1003G>A | p.Gly335Arg | Vip  Blood 2014 | DEL  (0) | PROB DAM  (0.996) | No | 1 |
|  |  | 19:  12995787 | G>C | 206/448  (46.3%) | c.1001C>G | p.Thr334Arg | Gallienne  2012 | DEL  (0) | PROB DAM  (0.982) | No | 1 |
| Patient 39  (P39) | *CDAN1* | 15:  43017748 | G>A | 1143/1162  (99.5%) | c. .3389C>T | p.Pro1130Leu | Dgany  2002 | DEL  (0) | PROB DAM  (0.999) | AA=0/  AG=1/  GG=6501 | 1 |
| Patient 40  (P40) | *CDAN1* | 15:  43018588 | G>A | 10/10  (100%) | c.3124C>T | p.Arg1042Trp | Dgany  2002 | DEL  (0) | PROB DAM  (0.993) | No | 1 |
| Patient 44  (P44) | *RPS19* | 19:  42365265 | G>A | 279/653  (43.1%) | c.156G>A | p.Trp52Ter | Willig  1999 |  |  | No | 1 |
| Patient 49  (P49) | *CDAN1* | 15:  43020440 | C>A | 256/544  (47.1%) | c.2830G>T | p.Ala944Ser |  |  | PROB DAM  (1.0) | No | 3 |
|  |  | 15:  43022955 | G>A | 167/324  (51.7%) | c.2015C>T | p.Pro672Leu | Dgany  2002 |  | PROB DAM  (1.0) | AA=0/  AG=3/  GG=6483 | 1 |
|  |  | 15:  43026447 | C>G | 483/969  (49.9%) | c.1234G>C | p.Ala412Pro |  |  | PROB DAM  (1.0) | No | 3 |
| Patient 50  (P50) | *CDAN1* | 15:  43019937 | A>  ACTGCTGC | 482/1179  (41.8%) | c.2971_2977  dupGCAGCAG | p.Val993Gly  fsTer13 |  |  |  | No | 2 |
|  |  | 15:  43022955 | G>A | 772/1478  (52.3%) | c.2015C>T | p.Pro672Leu | Dgany  2002 | TOL  (0.44) | PROB DAM  (0.999) | AA=0/  AG=3/  GG=6483 | 1 |
| Patient 51  (P51) | *CDAN1* | 15:  43028917 | G>A | 388/392  (99.2%) | c.152C>T | p.Pro51Leu |  |  | POSS DAM  (0.933) | No | 3 |
| Patient 53  (P53) | *CDAN1* | 15:  43022848 | GAAC>G | 3913/3980  (98.7%) | c.2125-2127del | p.Leu709del |  |  |  | No | 3 |
| Patient 54  (P54) | *PKLR* | 1:  155263320 | T>C | 1747/3425  (51.1%) | c.1178A>G | p.Asn393Ser |  | DEL  (0.02) | DEL  (0.02) | No | 3 |
|  |  | 1:  155261586 | CAG>CA | 576/1082  (53.2%) | c.1574Gdel | p.Ala526Glufs*3 |  |  |  |  | 2 |
| Patient 55  (P55) | *CDAN1* | 15:  43019890 | C>CAA | 362/672  (54.3%) | c.3024_3025  insTT | p.Glu1009Leu  fsTer24 | Tamary  2005 |  | PROB DAM  (0.994) | No | 1 |
|  |  | 15:  43028913 | G>C | 117/249  (46.9%) | c.156C>G | p.Phe52Leu | Tamary  2005 |  |  | No | 3 |
| Patient 56  (P56) | *CDAN1* | 15:  43022955 | G>A | 772/1478  (52.3%) | c.2015C>T | p.Pro672Leu | Dgany  2002 | TOL  (0.44) | PROB DAM  (0.999) | AA=0/  AG=3/  GG=6483 | 1 |
|  |  | 15:  43028913 | G>C | 117/249  (46.9%) | c.156C>G | p.Phe52Leu | Tamary  2005 |  |  | No | 3 |

The gene, genomic location, details of the variant, depth coverage of both reference and variant calls is shown for each gene as well as the cDNA and protein locations of the variants, the references where the Tier 1 variants were first identified, the predicted pathogenicity based on SIFT and Polyphen2 algorithms, the frequency of all the genotypes in the EVS (Exome variant server) and the Tier of the variant as per ACMG guidelines. The SIFT algorithm calculates the probability of a mutation having a deleterious effect (DEL) with a cut-off value of 0.05 (<0.05 = more likely deleterious than not, >/= 0.05= likely tolerated (TOL)). Polyphen-2 uses a prediction confidence calculation. A “probably damaging” (PROB DAM) mutation has a score >0.85 (false positive <10%), possibly damaging (POSS DAM) is 0.15-0.85 (false positives <18%) and the rest are classified as benign. SIFT and polyphen scores are given for each mutation alongside the effect. Genomic locations are given as chromosome:base location according to the HG19 build of the Genome. Read depth is given as reads with variant allele/total reads and %. cDNA and protein nomenclature follows HGVS guidelines.

**Supplementary Table IV:** Mutations of uncertain significance identified in samples from 3 patients analysed on the ORCP

| **Patient ID** | **Working diagnosis** | **Genes affected** | **Amino acid change (or splice site)** | **Reference/Tier** | **Patient progress** | **Comments** |
| --- | --- | --- | --- | --- | --- | --- |
| Patient 10  (P10) | Unexplained anaemia | *TERT* | p.Pro908LeufsTer2 | Tier 2 | Eventually diagnosed with SIFD (syndrome of sideroblastic anaemia, immune deficiency, fever and developmental delay) and underwent a bone marrow allograft. | Final diagnosis: SIFD (syndrome of sideroblastic anaemia, immune deficiency, fever and developmental delay) due to compund heterozygosity for *TRNT1* mutations. *TERT* mutation of uncertain significance as phenotype explained by the *TRNT1* mutations. |
| Patient 12  (P12) | Unexplained anaemia | *TERT* | p.Ala279Thr | Yamaguchi H 2005 | Several months after referral for investigation, repeat bone marrow aspiration confirmed aplastic anaemia and the patient underwent a bone marrow allograft. | Clinical team made aware of the presence of this TERT mutation, and this was taken into consideration in the management. As the data on the pathogenicity of this mutation is conflicting as it is reported as “probably damaging” in both the Exome Variant Server and the dbSNP database yet is reported as being present in ~4% of the population, the clinical team proceeded with a myeloablative conditioning regimen. |
| Patient 13 (P13) | Unexplained anaemia | *SEC23B* | p.Arg495His  p.Arg550Gln | Tier 3  Tier 3 | Possible new diagnosis of CDA-2 | BM morphology consistent, each parent shown to carry one allele. However, p.Arg495His was also found to be homozygous in a patient with G6PD deficiency in whom there is no evidence of CDA, casting doubt on the pathogenicity of this mutation. |
